# Surface Functionalization of Orthopedic Titanium Implants with Bone Sialoprotein

**DOI:** 10.1371/journal.pone.0153978

**Published:** 2016-04-25

**Authors:** Andreas Baranowski, Anja Klein, Ulrike Ritz, Angelika Ackermann, Joris Anthonissen, Kerstin B. Kaufmann, Christian Brendel, Hermann Götz, Pol M. Rommens, Alexander Hofmann

**Affiliations:** 1 Department of Orthopedics and Traumatology, University Medical Centre, Johannes Gutenberg University, Mainz, Germany; 2 Princess Margaret Cancer Centre, University Health Network, Toronto, Ontario, Canada; 3 Division of Pediatric Hematology/Oncology, Boston Children’s Hospital, Harvard Medical School, Boston, Massachusetts, United States of America; 4 Platform for Biomaterial Research, University Medical Centre, Johannes Gutenberg University, Mainz, Germany; University of Texas Southwestern Medical Center, UNITED STATES

## Abstract

Orthopedic implant failure due to aseptic loosening and mechanical instability remains a major problem in total joint replacement. Improving osseointegration at the bone-implant interface may reduce micromotion and loosening. Bone sialoprotein (BSP) has been shown to enhance bone formation when coated onto titanium femoral implants and in rat calvarial defect models. However, the most appropriate method of BSP coating, the necessary level of BSP coating, and the effect of BSP coating on cell behavior remain largely unknown. In this study, BSP was covalently coupled to titanium surfaces via an aminosilane linker (APTES), and its properties were compared to BSP applied to titanium via physisorption and untreated titanium. Cell functions were examined using primary human osteoblasts (hOBs) and L929 mouse fibroblasts. Gene expression of specific bone turnover markers at the RNA level was detected at different intervals. Cell adhesion to titanium surfaces treated with BSP via physisorption was not significantly different from that of untreated titanium at any time point, whereas BSP application via covalent coupling caused reduced cell adhesion during the first few hours in culture. Cell migration was increased on titanium disks that were treated with higher concentrations of BSP solution, independent of the coating method. During the early phases of hOB proliferation, a suppressive effect of BSP was observed independent of its concentration, particularly when BSP was applied to the titanium surface via physisorption. Although alkaline phosphatase activity was reduced in the BSP-coated titanium groups after 4 days in culture, increased calcium deposition was observed after 21 days. In particular, the gene expression level of RUNX2 was upregulated by BSP. The increase in calcium deposition and the stimulation of cell differentiation induced by BSP highlight its potential as a surface modifier that could enhance the osseointegration of orthopedic implants. Both physisorption and covalent coupling of BSP are similarly effective, feasible methods, although a higher BSP concentration is recommended.

## Introduction

Most of the implants currently used in orthopedic surgery are composed of titanium or cobalt–chromium alloys [1]. Titanium (Ti) displays a combination of exceptional biocompatibility with elasticity [2,3] and the ability to integrate into local bone stock [4–7], and these properties render Ti as optimal for permanent implantation. Despite recent developments in the generation of new alloys and in biomaterial surface modifications, 10% of replaced joints require surgical revision within 15 years [8]. In an aging population with a growing need for prosthetic joint replacement, reducing complications that lead to revision surgery is of the utmost importance [9–11]. Micromotion at the bone-implant interface is responsible for implant loosening, which is often requires revision surgery. Phagocytosis of the resulting wear debris by macrophages leads to a pro-inflammatory response along with increased stimulation and generation of osteoclasts [12]. These events cause bone resorption to outweigh bone formation at the bone-implant interface and induce the generation of a fibrous capsule [13–16].

Traditionally, this problem has been addressed by developing inert materials that decrease the host’s immune response to the implant. In the last several years, many attempts have been made to activate the implant surface by changing its topography and morphology using mechanical and physiochemical methods. The aim of these modifications was to accelerate osseous anchorage and bone ingrowth, thereby reducing micromotion at the interfacial zone. In addition, inorganic coatings that mimic the mineral phase in natural bone and subsequently enhance bone ingrowth and implant stability were developed [17,18].

In the field of bone tissue engineering (BTE), various biomaterials have been tested to determine their suitability as biomimetic scaffolds or implant coatings that promote homing of osteogenic cells, differentiation of progenitor cells, and early vascular ingrowth. The multitude of biomaterial families includes calcium phosphate (CaP)-based ceramics/coatings and their composites, such as the combination of these CaP-based materials with polymers such as poly(D,L-lactic-co-glycolic acid) (PLGA), chitosan, organic collagen fibers or gelatin [19]. As natural bone is a composite material, an obvious biomimetic option as a BTE material is the combination of both inorganic and organic phases. Combining natural biopolymers such as bacterial cellulose, collagen or chitosan with inorganic hydroxyapatite can produce a nanocomposite material with excellent biodegradability and bioactivity. However, challenges of clinical translation remain due to deficient vascular ingrowth, inadequate control of bone infections and the insufficient mechanical strength of scaffolds mimicking the biological functions of native osteoid [20].

Another branch of the biomaterial family consists of moldable hydrogels of natural (e.g., collagen, gelatin) and synthetic origin (e.g., poly(ethylene glycol), poly(vinyl alcohol)); these materials are particularly appreciated for their biocompatibility and biodegradability. Hydrogel matrices can be solidified via incorporation of (in)organic nanoparticles such as hydroxyapatite, clay, metal or graphene, thus forming nanocomposite hydrogels (NC gels), or can stiffen upon injection via interactions with body fluids (e.g., self-assembling RAD16-I peptides) [21,22].

Wang et al. created cohesive colloidal gels of differently charged nanoparticles. PLGA can be coated with the natural polymer chitosan to create a positively charged nanoparticle that is capable of forming a porous network with negatively charged nanoparticles such as hydroxyapatite or PLGA-alginate. The resulting colloidal gels show promise in terms of their shape retention, their response to shear force and their established osteoconductivity in a rat calvarial defect model [23–26]. A possible field of application of hydrogels is their use as a biomimetic tissue engineering scaffold, either as an implant coating or as an injectable hydrogel that is able to fill irregular shaped defects of the bone via minimally invasive surgery [19]. Another method of reducing micromotion via osseous anchorage and bone ingrowth at the interfacial zone involves coating the implant with proteins found in the extracellular organic matrix (ECM) of bone. Under untreated conditions, the first layer of proteins on an implant consists of unselected adsorbed blood serum proteins and interstitial fluids that may trigger an inflammatory response [27,28]. By coating the surface with ECM proteins, the initial immune response can be modified because these proteins represent cell adhesion motifs and may function as scaffolds for bone cells. Bone sialoprotein (BSP) is a tissue-specific phosphoprotein of the ECM that combines the functions of its cell-binding RGD motif, the properties of a nucleating motif for mineralization, and the ability to promote angiogenesis [29,30]. Its RGD motif interacts with the α_ν_β_3_-receptor (vitronectin receptor) of osteoblasts and regulates the adhesion and differentiation of osteoprogenitor cells, which are known to be critical for tissue mineralization [29,31–33]. BSP induces hydroxyapatite cluster formation via its nucleating motif [34]. Several authors have previously described the function of BSP on different surfaces [31,35–40]. However, the best method for coating BSP onto a surface and the necessary levels of BSP remain unclear. O’Toole used a gelatin film as a carrier medium for BSP, and this formulation promoted new bone formation but showed poor mechanical resistance in pullout tests [41]. Unpublished tests of simple protein adsorption on untreated titanium revealed an uneven distribution, weak binding, and rapid and uncontrolled desorption of the protein. The aim of this study was to compare the physisorption and covalent coupling methods of applying BSP at different concentrations to an activated titanium surface in terms of the influence of these materials on primary human osteoblasts. We hypothesized that BSP influences osteoblast behavior in a concentration-dependent manner by attracting osteoblastic cells, upregulating osteoblast-specific gene expression, accelerating cell differentiation, and enhancing mineralization of the ECM.

## Materials and Methods

### Materials

Pure grade IV titanium disks (1.6 mm thick with either a 10.5 mm or 33.5 mm diameter) were obtained from Medartis^®^ (Basel, Switzerland). BMP-7 was purchased from Miltenyi Biotech (Bergisch Gladbach, Germany). All other materials were obtained from Sigma-Aldrich^®^ (Taufkirchen, Germany) unless otherwise indicated.

### Human recombinant bone sialoprotein

All experiments were conducted using human recombinant BSP provided by Immundiagnostik AG (Bensheim, Germany). The recombinant BSP was produced by a stable Chinese hamster ovary (CHO) cell line.

### Cell culture

Primary human osteoblasts (hOBs) were isolated from human bone specimens acquired during hip or knee joint replacement surgeries. The patients provided written consent for the use of the residual material. All research was approved by the ethics committee of the Landesärztekammer Rheinland-Pfalz in accordance with the principles expressed in the Declaration of Helsinki and the ICH Guidelines for GCP.

The cells were isolated according to a previously described protocol [42,43]. Cancellous bone fragments were washed with PBS, followed by digestion with collagenase type I (Worthington Biochemical Corporation, NY, USA) for 45 minutes at 37°C. After another step of washing with PBS, the cells were distributed in 6-well tissue culture plates (Greiner Bio-One, Frickenhausen, Germany) and cultured in DMEM/F12 medium (Gibco^®^, Life Technologies, Grand Island, NY, USA) supplemented with 20% fetal calf serum (Biochrom AG, Berlin, Germany) and 1% penicillin and streptomycin (Gibco^®^, Life Technologies, Grand Island, NY, USA). After the first passage, the hOBs were cultured in DMEM/F12 medium supplemented with 10% fetal calf serum and 1% penicillin and streptomycin. Media were changed two times per week. The differentiation medium also contained 10^−8^ M dexamethasone, 3.5 mM ß-glycerophosphate, and 50 μg/ml ascorbic acid. L929 fibroblasts (Promocell, Heidelberg, Germany) were cultured in MEM with Earles salts (Biochrom AG, Berlin, Germany) supplemented with 10% fetal calf serum and 1% penicillin and streptomycin. All cells were cultured in a humidified atmosphere containing 5% CO_2_ at 37°C. For the seeding efficiency and cell visualization experiments, hOBs were transduced once with eGFP using a lentiviral SEW-eGFP vector according to a standard protocol [44,45]. The transduction efficiency was determined by flow cytometry (Accuri^®^ C6 Flow Cytometer, BD Biosciences, San Jose, CA, USA).

### Preparation of titanium disks

All titanium disks, except for the untreated controls, were cleaned, and their surfaces were activated overnight in piranha solution (a mixture of 1 part 30% H_2_O_2_ solution (Merck, Darmstadt, Germany) and 4 parts concentrated sulfuric acid (Merck, Darmstadt, Germany)). After this step, the disks were washed with sterile water.

**Fig 1:**
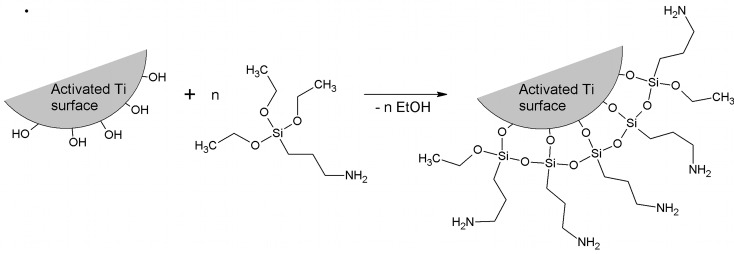
Functionalization of an activated titanium surface using (3-aminopropyl)triethoxysilane (APTES) linkers.

The process of covalently coupling BSP to the titanium disks began with an incubation step for 30 minutes in a 1% aminosilane (APTES) solution (Fig 1), followed by cleaning with sterile water. A subsequent incubation for 1 hour with 5% glutaraldehyde solution (AppliChem GmbH, Darmstadt, Germany) was performed to bind the silane to glutaraldehyde. Finally, BSP solution was added, and the mixture was incubated for 1 h.

Alternatively, physisorption of BSP to the titanium disks was achieved by activating the surface using piranha solution, rinsing the disks with water, and directly incubating the disks for 1 hour in BSP solution. After the coupling and adsorption processes, the disks were washed two times with PBS and stored in PBS until use. Untreated titanium implants served as controls.

### Surface analysis

The elemental composition of the surface (focusing on nitrogen (N), carbon (C), oxygen (O) and silicon (Si)) was analyzed via x-ray photoelectron spectroscopy (XPS). Samples from every step of the coupling process were examined using the PHI 5600-CI XPS system (Physical Electronics, Eden Prairie, MN, USA).

### Cell viability

The viability of cells cultured on titanium disks coated with BSP at two different concentrations (50 μg/ml diluted or 280 μg/ml undiluted BSP solution) via either covalent coupling or physisorption was investigated using a well-established 3-(4,5-dimethylthiazol-2-yl)-2,5-diphenyltetrazolium bromide (MTT) assay [46]. This colorimetric assay is a standardized measure of cytotoxicity. In deviation from the German Industry Norms (DIN) of ISO 10993–5:2009, we seeded L929 fibroblasts onto the titanium disks in 24-well plates. Untreated titanium disks served as internal controls, and 0.1% ZDEC polyurethane films (A-131) served as negative controls. L929 fibroblasts were seeded at a density of 60,000 cells per disk on prepared titanium disks (⌀ 10.5 mm) that were placed in ultra-low-attachment 24-well plates (Corning Life Sciences, NY, USA), and the cells were incubated for 24 hours. Subsequently, the media were removed, 300 μl of 1 mg/ml MTT solution were added, and the cells were incubated for an additional 2 hours. The MTT solution was then replaced with 600 μl of isopropanol. The absorption of the resulting solution was measured at 570 nm (reference 650 nm) using a spectrophotometer (Tekan, Sunrise, Maennedorf, Switzerland).

### Seeding efficiency

Adhesion of hOBs to untreated titanium and to titanium coated with BSP (280 μg/ml BSP solution) via covalent coupling or physisorption was investigated. Ultra-low-attachment plates served as negative controls. A total of 50,000 eGFP-transduced cells were seeded onto each prepared titanium disk (⌀ 10.5 mm), and these disks were placed in an ultra-low-attachment 24-well plate (Corning Life Sciences, NY, USA). After 1, 4, and 24 hours, the medium was aspirated. Non-adherent cells were removed, and the disks were washed with PBS. The fluorescence intensity of the adherent cells was measured using the GloMax^®^-Multi Detection System (filter with 490 nm extinction and 510–570 nm emission, Promega, Madison, WI, USA). The numbers of adherent cells were calculated using an established standard curve.

### Cell proliferation and cell visualization

The alamarBlue^®^ assay was used to analyze cell proliferation over a defined period of time. Compared to the MTT assay, the alamarBlue^®^ assay is non-cytotoxic and allows repeated measurements of a cell population [47,48]. hOBs (15,000 cells per disk) were seeded onto prepared titanium disks (⌀ 10.5 mm; untreated Ti or Ti coated with BSP (36 μg/ml and 280 μg/ml) via covalent coupling or physisorption, and the disks were placed in ultra-low-attachment 24-well plates (Corning Life Sciences, NY, USA). After 1, 4, 7, 10, and 14 days, the media were removed. The cells were incubated at 37°C for 4 hours with 500 μl of a 10% solution of alamarBlue^®^ (Invitrogen, Karlsruhe, Germany) in complete cell culture medium. Subsequently, the media were transferred into 96-well plates, and the fluorescence intensity was measured using the Glomax^®^-Multi Detection System (filter with 525 nm extinction and 580–640 nm emission, Promega, Madison, WI, USA). Cells seeded onto 24-well tissue culture plates (Cellstar^®^, Greiner Bio-One, Frickenhausen, Germany) served as positive controls. For negative controls, incubation media without cells were used.

In accordance with the cell proliferation assay, eGFP-transduced hOBs were seeded onto prepared titanium disks (coated with 50 μg/ml BSP solution). After 1, 3, and 5 days, cell proliferation was analyzed using a confocal laser-scanning microscope (Leica TCS SP2, Bensheim, Germany) and an EVOS^®^ fluorescence microscope. CLSM images were acquired using an argon-krypton laser (excitation at 488 nm, detection at 500–550 nm) using a water-immersion objective (20x magnification).

### Cell migration

Cell migration was assessed using a well-established Boyden chamber assay. For this assay, 24-well plates (Falcon^®^, Corning, NY, USA) were prepared. The prepared titanium disks (untreated titanium or titanium coated with either 50 or 280 μg/ml BSP solution via physisorption or covalent coupling) and 700 μl of medium (supplemented with 1% fetal calf serum) were added to the wells. In the control group, medium supplemented with 10% fetal calf serum was used. The inserts (Falcon^®^ cell culture inserts, pore size: 8 μm, Corning, NY, USA) were placed into the wells, and 90,000 hOBs were seeded onto the inserts. After 12 and 24 hours of incubation, the medium was removed, and the wells were washed two times with PBS. The cells located on the upper membrane of the inserts were removed with a cotton swab. The inserts were washed again with PBS, followed by incubation for 1 hour in cell dissociation buffer (Gibco^®^, Life Technologies, Grand Island, NY, USA) supplemented with calcein-AM (Molecular Probes^™^, Leiden, The Netherlands). Then, 100 μl of the solution were transferred to a black 96-well plate (Greiner Bio-One, Frickenhausen, Germany), and the fluorescence intensity was analyzed using the GloMax^®^-Multi Detection System (filter with 490 nm extinction and 510–570 nm emission, Promega, Madison, WI, USA). Cell numbers were calculated using an established standard curve.

### Alkaline phosphatase activity

hOBs and L929 fibroblasts (serving as negative controls) were seeded on prepared titanium disks (⌀ 33.5 mm disks: untreated titanium with proliferation media, titanium disks coated with BSP (50 μg/ml BSP solution) via physisorption, or titanium disks coated with BMP-7 (1 μg/ml, positive control) via physisorption). After 4 days of incubation, the cells were harvested. The alkaline phosphatase (ALP) activity level was determined via an Alkaline Phosphatase Assay (Abcam^®^, Cambridge, UK).

### Mineralization of the extracellular matrix

hOBs were cultured in cell culture media supplemented with either 1 mg/ml BSP or 100 ng/ml BMP-7 for 21 days. In the positive control group, the cells were incubated with differentiation medium. Non-mineralizing L929 fibroblasts served as negative controls. On day 21, the cells were fixed with Roti^®^ Histofix (Carl Roth, Karlsruhe, Germany) for 15 minutes and then washed several times with PBS. Cell layers were stained for 20 minutes with Alizarin Red S solution (pH 4.0) and washed again with sterile water. The cell layers were photographed with a Zeiss Axioskop microscope (10x objective). Optical microscopy images of Alizarin Red S-stained osteoblasts were processed using Image J software (Version 1.48, NIH) to create a binary presentation. When the intensity of Alizarin Red S staining reached a threshold value, the associated pixel was displayed as black in the binary image; when the staining intensity was below the threshold value, the associated pixel was displayed as white. In this manner, calcium deposition was depicted in black color to enhance the discriminatory power of the staining results and to facilitate quantitative analysis.

### Gene expression

For this experiment, pure titanium, titanium disks coated with BSP (30 μg/ml diluted or 280 μg/ml undiluted BSP solution) via either covalent coupling or physisorption, titanium disks activated with piranha solution, and titanium disks coated with BMP-7 (100 ng/ml) via physisorption were prepared. hOBs were seeded on the prepared titanium disks (⌀ 33.5 mm), which were placed in a 6-well ultra-low-attachment plate (Corning, NY, USA). After 1, 4, and 7 days, the cells were collected, and RNA was isolated using a Qiagen RNeasy Mini Kit (Qiagen GmbH, Hilden, Germany). RNA was transcribed using random hexamer primers (Promega, Madison, WI, USA) and Superscript RT III (Invitrogen, Carlsbad, CA, USA) in a thermocycler (PEQLAB Primus 96 advanced, PEQLAB Biotechnologie GmbH, Erlangen, Germany). For real-time polymerase chain reaction (RT-PCR), QuantiTect^®^ primers (Qiagen GmbH, Hilden, Germany) for 18S RNA (QT00199367), ALP (QT00012957), SP7 (QT00213514), type I collagen (Col1) (QT00037793), RUNX2 (QT00020517), osteopontin (OPN) (QT01008798), and SPARC (QT00018620) as well as SYBR Green PCR master-mix were used in a 7300 real-time PCR system (Applied Biosystems Deutschland GmbH, Darmstadt, Germany). The following thermal cycling profile was used: 2 minutes at 50°C, 10 minutes at 95°C, and 40 cycles of 15 seconds at 95°C, 30 seconds at 55°C and 35 seconds at 72°C. At the end of thermal cycling, a dissociation step was added as a control. RT-PCR results were calculated using the well-established 2^-ΔΔCt^ method, and the results were represented as the expression levels relative to the values for incubation on untreated titanium [49].

### Statistical analysis

Statistical analysis was performed using SPSS 22.0 software (SPSS Inc., Chicago, IL, USA). The results are depicted as the means ± standard deviation or as medians and quartiles. One-way ANOVA with Levene's test for equality of variances was performed, and either Tukey's honest significant difference (HSD) post hoc test or the Welch test with a Games-Howell post hoc test was performed if applicable. Triplicate measurements for each time point and experimental condition were performed. Differences corresponding to p<0.05 were considered statistically significant.

## Results and Discussion

### Surface composition

The surface of untreated titanium showed high contents of carbon (67%) and oxygen (31%) as well as low levels of titanium (0.9%, Table 1). This low titanium level is explained by the 3-7-nm-thick titanium dioxide layer covering the implant. The high carbon content of the surface indicates "normal" organic hydrocarbon contamination, which inevitably occurs when titanium is exposed to air [50]. Because this contamination can exert an uncontrollable influence on further surface coating and, subsequently, on seeded cells, the titanium samples in our study were cleaned with a sulfuric acid/hydrogen peroxide mixture (piranha solution) before BSP coating was performed. This method enables controlled oxidation of the titanium surface, creating stable conditions for further modifications [50,51]. The increase in the titanium content to 18% and of the oxygen content to 52% as well as the associated decrease in the carbon content to 25% after oxidation reflected a reduction in organic impurities and formation of a hydrated titanium oxide layer due to treatment with piranha solution (Table 1). Another effect of this treatment was increased roughness of the surface, which promotes cell adhesion and proliferation [52–54].

**Table 1:** Surface elemental composition (percentage) of untreated and piranha-oxidized titanium surfaces as determined by XPS.

	Ti [%]	C [%]	N [%]	O [%]
Ti untreated	0.9	67.0	0.5	31.1
Piranha-treated Ti	18.2	25.6	1.1	52.3

### Physisorption and covalent coupling

In addition to the surface topography, the osseointegration of an implant can be markedly affected by bioactive protein coatings [37,55]. The bioactive protein BSP may promote osteoblast adhesion via RGD-mediated interactions and may induce an increase in mineralization of the ECM by stimulating the differentiation of these cells [32,55,56]. Physisorption represents a simple and effective method of surface coating. One theoretical disadvantage of this type of protein adsorption is the uncontrolled release of the protein, which may reduce its concentration at the bone-implant interface due to diffusion, thus causing unwanted systemic effects. To identify the best coating method, we compared physisorption with a covalent coupling method.

After several pre-tests, APTES (covalent bonding via (APTES) and a glutaraldehyde linker) was chosen because it bound the greatest amount of BSP to the titanium surface compared to other covalent coupling agents, including 3-aminopropyltrimethoxysilan (APTS) and phosphate (data not shown). APTES offers the advantage of strong coupling of BSP, which restricts BSP desorption and restrains the effects of BSP to the interface. The glutaraldehyde linker ensures that the coupled molecules remain a certain distance from the surface, preventing conformational rearrangements and providing greater mobility and accessibility to adhering cells [50].

Samples collected at different coating steps were evaluated via XPS. In the group of disks coated with BSP via physisorption, the contents of carbon and nitrogen increased due to the carbon skeleton and N-containing groups of BSP. In the first step of the covalent coupling process, the APTES-treated titanium surface contained higher silicon and nitrogen contents than the untreated titanium surface. After the addition of the glutaraldehyde linker, the carbon content increased due to the additional pentyl group, and the silicon content of the surface was consequently reduced. In the final coupling step, BSP was covalently coupled to the glutaraldehyde linker. Due to the numerous N-containing groups of BSP, a further increase in the nitrogen content was detected (Table 2).

**Table 2:** Surface elemental composition (percentage) after surface coating with BSP (280 μg/ml) and covalent coupling via APTES and glutaraldehyde (GA) as a linking agent.

	Ti [%]	C [%]	N [%]	O [%]	Si [%]
Physisorption (Ti + BSP)	12.1	41.9	3.4	40.3	1.0
Ti + APTES	10.6	42.9	2.9	38.2	4.5
Ti + APTES + GA	8.2	46.2	3.4	36.4	4.0
Ti + APTES + GA + BSP	5.8	51.9	7.1	31.3	2.9

### Cell viability

**Fig 2:**
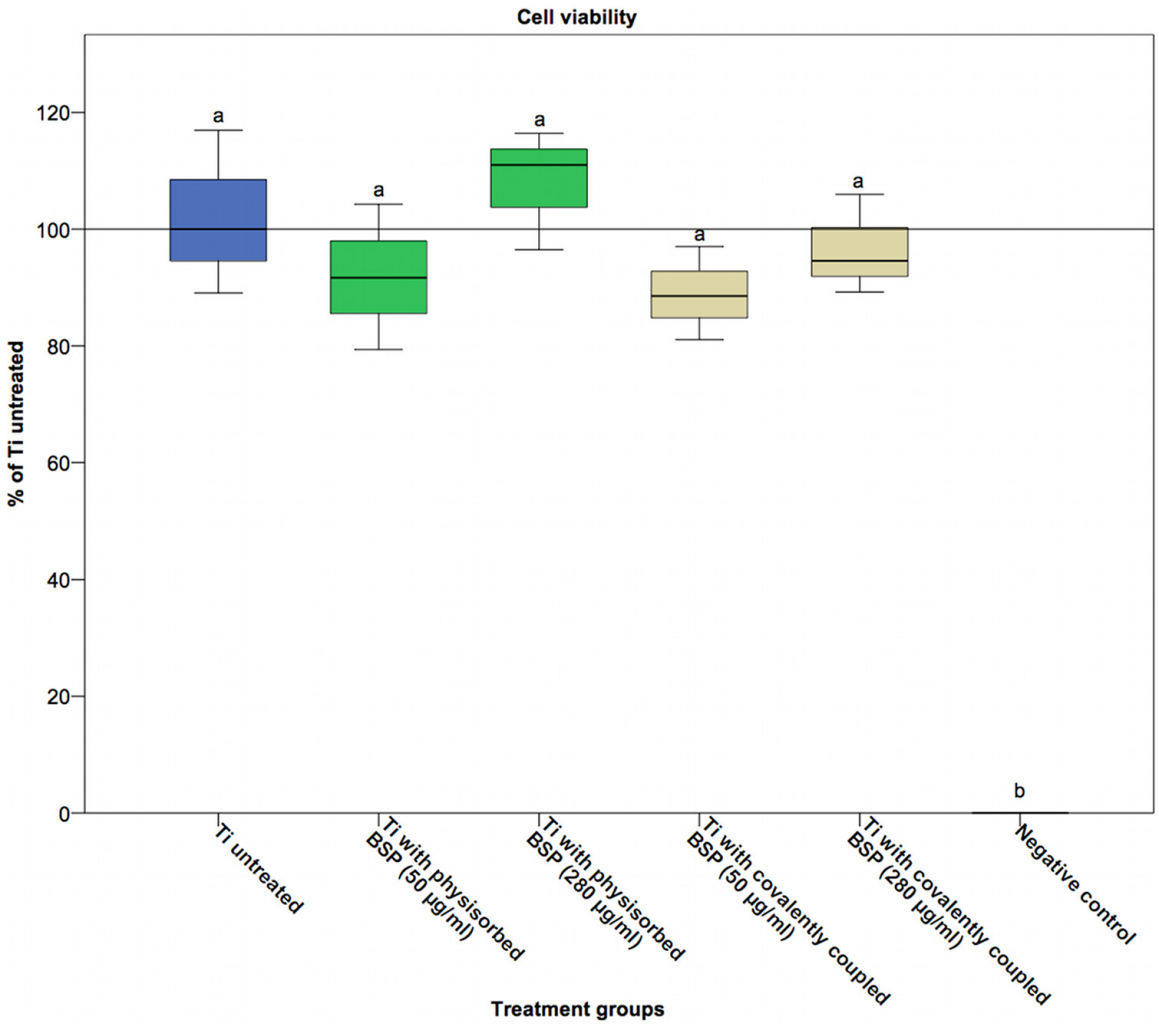
Viability of cells cultured on BSP-treated titanium. Titanium coated with 50 μg/ml or 280 μg/ml BSP via either physisorption or covalent coupling did not show a statistically significant difference in cell viability compared to untreated titanium. In the negative control group (0.1% ZDEC polyurethane, A-131), no viable cells were detected after 24 hours (p<0.01). Statistically significant differences (p<0.05) between the groups are indicated by different letters.

Analysis of cell viability was performed using a well-established MTT assay. Each BSP coating method was examined at two different BSP concentrations (50 μg/ml and 280 μg/ml). We found that the modified titanium surfaces did not exert any toxic effects on cells regardless of the BSP concentration (Fig 2). The percentages of viable cells that were cultured on BSP-coated Ti disks were 87 ± 1.9% (50 μg/ml group) for covalent coupling and 106 ± 2.3% (280 μg/ml group) for physisorption relative to the percentage of viable cells cultured on untreated titanium (median set as 100%).

### Seeding efficiency

**Fig 3:**
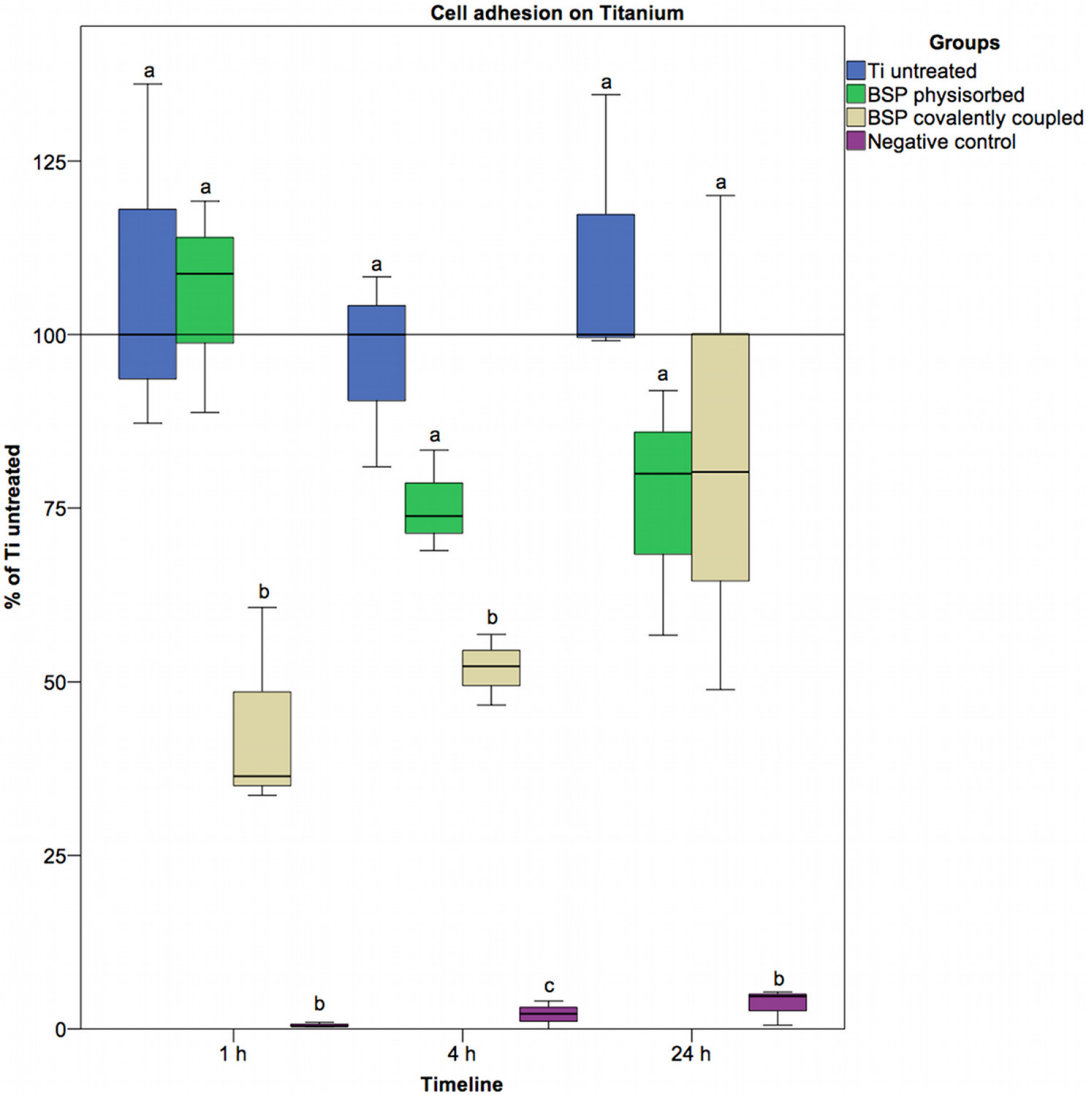
Results of the seeding efficiency assay (coating with 280 μg/ml BSP solution). Statistically significant differences (p<0.05) are indicated by different letters.

The initial attachment of cells to the surface of a material is a basic requirement for further biological processes, such as cell survival, activation, proliferation, differentiation, and migration [57]. For the cell adhesion test, we removed the incubation media and the non-adherent cells at different time points after cell seeding, and the remaining cells were subjected to several rinses. If a cell was not removed from the surface by rinsing, it was considered adherent [58]. Compared with the untreated titanium surface, the APTES-BSP-surfaces contained significantly fewer adherent cells (Fig 3). These initial differences diminished with increasing incubation time. After 24 hours of incubation, no statistically significant difference in adherent cells was detected between the sample groups. One possible explanation for this observation might be the shielding effect of the alkyl silane layer. This layer is impermeable to peptides and proteins and may therefore decrease the amount of BSP that is non-specifically bound to the titanium surface via physisorption. In addition, direct contact of cells with the titanium oxide layer is hampered by the overlying alkyl silane layer [50,59,60]. By incorporating phosphate ions into the oxide layer and forming a calcium phosphate layer that resembles an apatite layer, direct cell contact to this zone may accelerate adhesion [61–63]. The oxide layer itself appears to promote osseointegration [64]. Cell adhesion to titanium surfaces treated with BSP via physisorption was not significantly different from that to untreated titanium at any time point. The tendency toward a reduction in cell adhesion in the physisorption group could be explained by the gradual diffusion of BSP from the titanium surface, leading to saturation of the integrin receptors of the cells. Experiments using free RGD motifs have shown that the adhesion of osteoblastic cells can be reduced in this fashion [65].

### Cell proliferation

**Fig 4:**
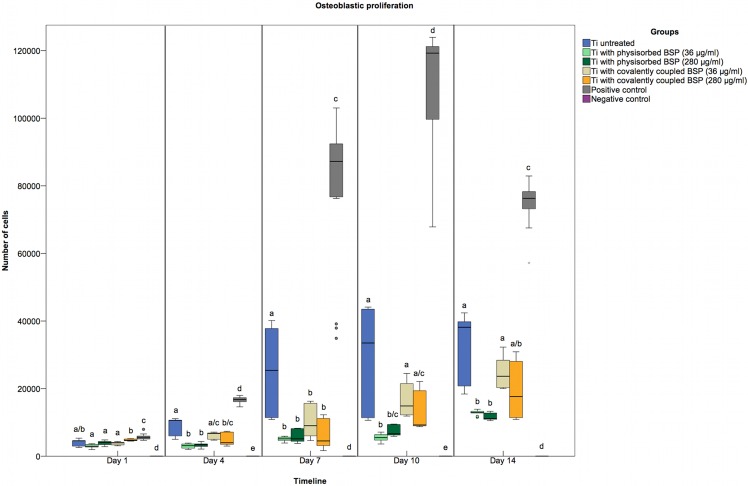
Osteoblastic proliferation on untreated and BSP-coated titanium. BSP at a concentration of either 36 or 280 μg/ml was coated onto titanium disks via covalent coupling or physisorption. As a positive control, cells were seeded onto tissue culture plates. As a negative control, cell-free samples were used. hOBs proliferated significantly faster on untreated titanium than on titanium coated with BSP via covalent coupling (during the first seven days) and via physisorption (throughout the 14-day observation period). Statistically significant differences (p<0.05) between distinctly treated samples are indicated by different letters.

The alamarBlue^®^ assay was used to assess the proliferation of primary hOBs on various modified materials over time. During the first week of incubation, hOBs proliferated more rapidly on untreated titanium than on BSP-coated titanium (Fig 4). Cell proliferation in the BSP physisorption groups remained significantly lower than that in the untreated titanium groups. In the APTES-BSP groups, however, no significant differences in cell proliferation compared to the untreated titanium groups were observed after day 7. Cells cultured on disks coated with BSP via covalent coupling proliferated more rapidly than those cultured on disks coated with BSP via physisorption. The difference between the two groups was greater at the lower concentration of BSP (36 μg/ml). These results are consistent with data available in the literature that show an accelerated osteogenic differentiation rate, a reduced proliferation rate and a higher apoptosis rate in cells overexpressing BSP [66]. This finding could be because increased osteogenic activity is generally accompanied by decreased cell proliferation [67–69].

### Cell morphology

**Fig 5:**
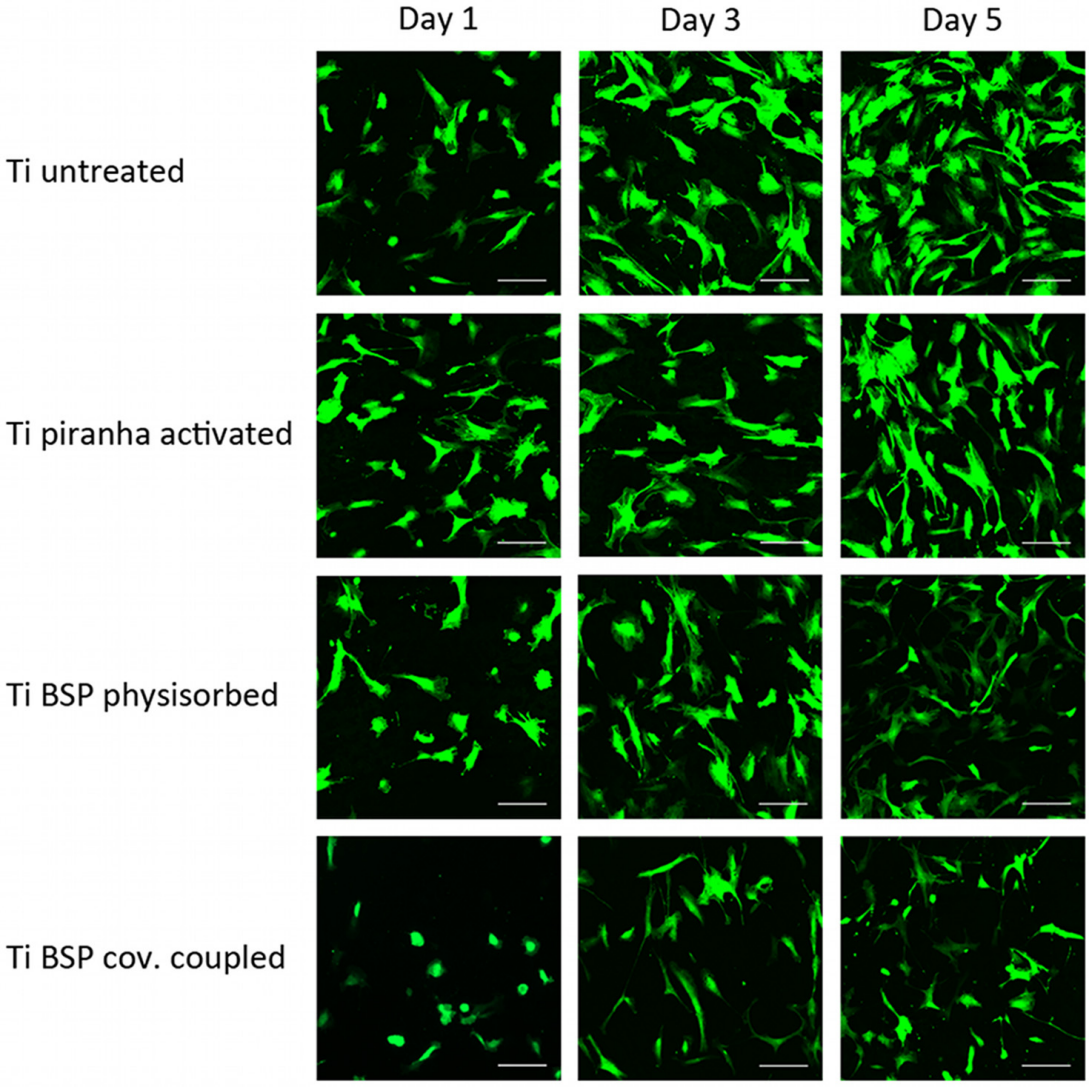
hOBs cultured for 1, 3, and 5 days on untreated Ti, piranha-treated Ti, BSP (50 μg/ml)-coated Ti via physisorption, and APTES-BSP (50 μg/ml)-Ti showed reduced cell spreading and confluency on BSP-treated surfaces compared to untreated or piranha-treated Ti (scale bars were 150 μm).

To determine the morphology of hOBs cultured on distinctly treated titanium surfaces, eGFP-transduced hOBs were analyzed via CLSM after 1, 3, and 5 days in culture. On day 1, a greater number of cells with a spherical shape appeared on the BSP-coated titanium surfaces than on the uncoated surfaces (Fig 5). Accordingly, cell spreading, as well as the confluency of the total cell population, was reduced on the BSP-coated surfaces. No differences in cell morphology or in the confluency of the cell layer was observe untreated titanium surfaces and titanium surfaces that were activated with piranha solution. The surface structure has been described to impact cell morphology. Surface roughness remained unaffected by the APTES coating [70]. Because the two BSP coating methods resulted in a similar cell morphology and cell density, the observed effects of BSP coating on cells was likely caused by BSP. Hilbig also described a spherical cell morphology for cells cultured on BSP-coated titanium with hydroxyapatite ceramic (TICER); the morphology of these cells became polygonal after 24 hours, and this morphology is typical for adherent osteoblasts [39].

### Cell migration

**Fig 6:**
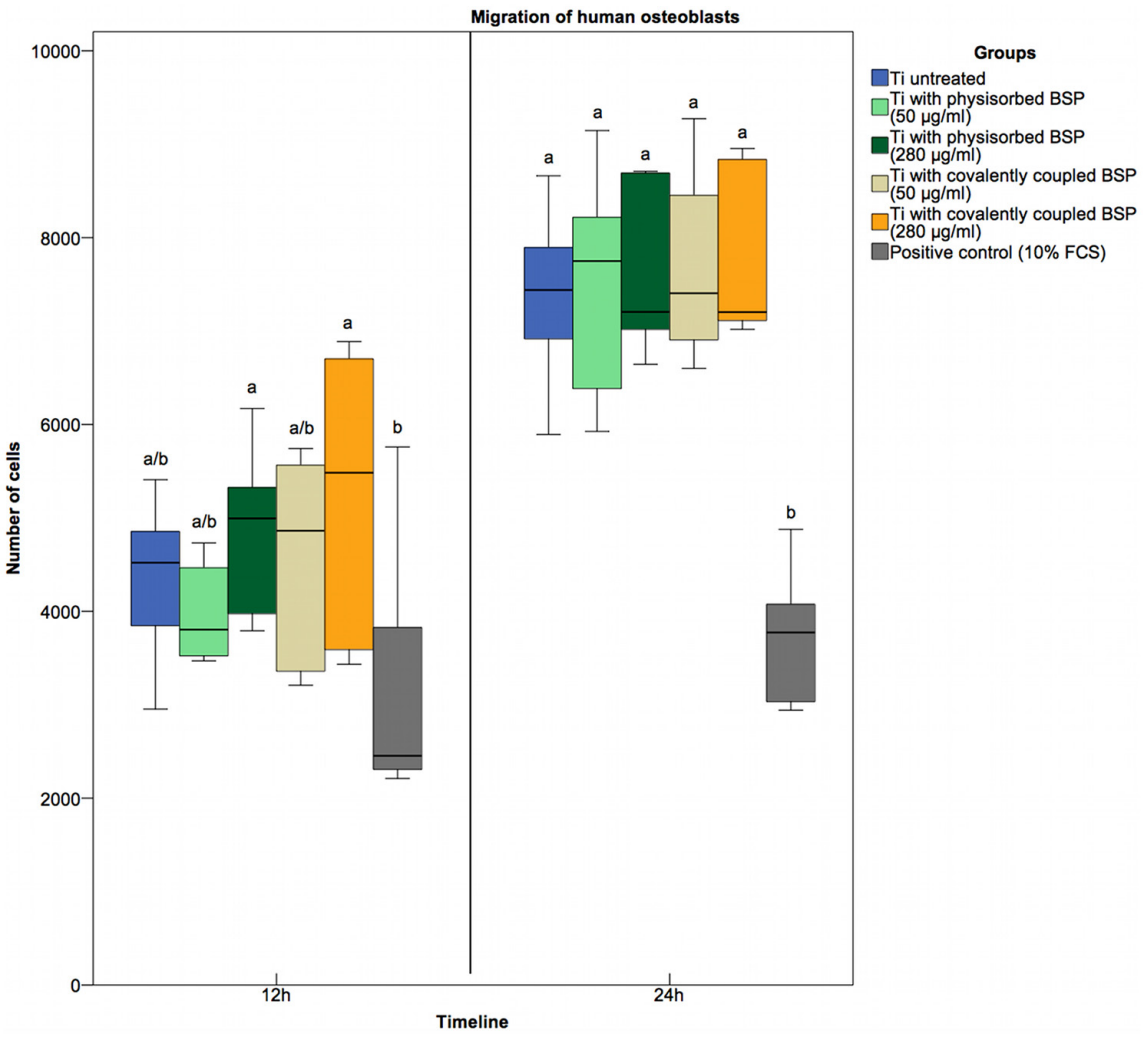
Human osteoblast migration after 12 and 24 hours. From left to right: untreated titanium as a control, BSP-coated Ti via physisorption (coated with 50 or 280 μg/ml BSP solution), APTES-BSP-Ti (coated with 50 or 280 μg/ml BSP solution), and positive control (medium + 10% FCS). After 12 hours, only 280 μg/ml BSP-coated Ti via physisorption and APTES-BSP-Ti (280 μg/ml BSP) showed significantly higher rates of hOB migration than the control surface. After 24 hours, all titanium samples showed higher hOB migration rates than the control. Statistically significant differences (p<0.05) at 12 hours and 24 hours are indicated by different letters.

Chemotaxis and migration of cells to a positive stimulus can simulate the recruitment of local cells in an organism. To determine the influence of BSP on the migration of osteoblasts, a Boyden chamber assay was used. After 12 hours, a tendency toward greater cell numbers on titanium disks treated with the higher concentration of BSP solution (280 μg/ml) compared to untreated titanium disks and titanium disks treated with the lower BSP concentration (50 μg/ml) was found. The number of migrated cells in both “high concentration” groups was significantly greater than that in the control group, which was treated with 10% FCS (Fig 6). After 24 hours, there was no difference in cell migration between the “high concentration” groups and the other groups. All titanium samples showed twofold higher cell migration than the 10% FCS control-treated samples. Byzova provided evidence that BSP exerts a strong chemotactic effect on UMR 106–01 osteosarcoma cells [65]. After additional stimulation of α_ν_β_3_ integrins using external agonists (PMA, ADP, etc.), the rate of cell migration was only slightly increased; this result indicated that in UMR 106–01 cells, α_ν_β_3_ integrins were already in a highly activated state and therefore enabled optimal recognition of BSP. In this study, no strong chemotactic effect of BSP on primary hOBs was observed. hOBs were likely in a fully differentiated and partly senescent state, and therefore, excessive stimulation of cell responses was less likely in hOBs than in UMR 106–01 cells. Osteoblast-like cells or osteoblast-like cell lines are heterogeneous with respect to their phenotypic characteristics and gene expression profiles [71]. Thus, the results of Byzova´s study can be explained by differences in the density or degree of activation of α_ν_β_3_ integrins.

### Activity of alkaline phosphatase and mineralization of the extracellular matrix

**Fig 7:**
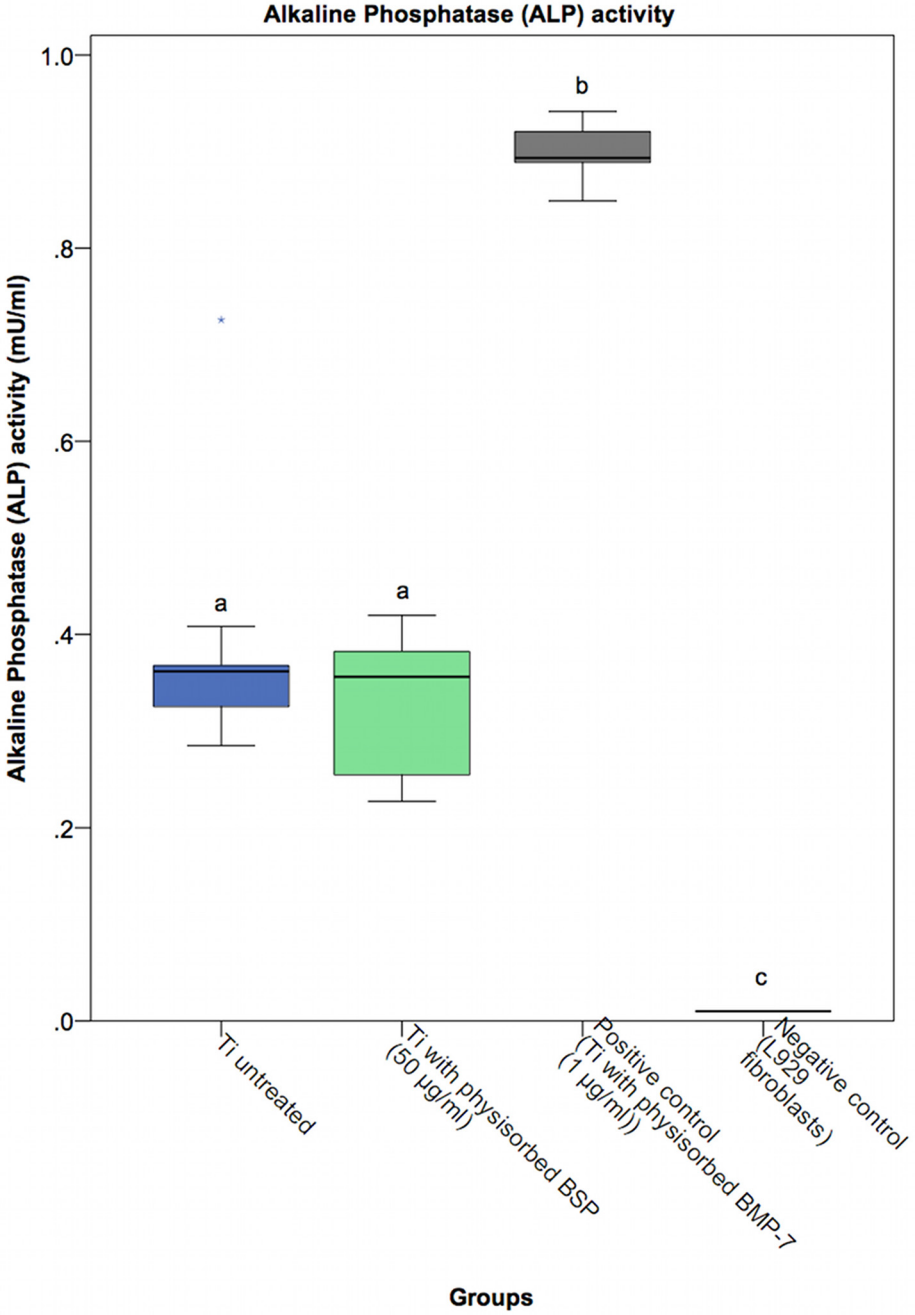
ALP activity of osteoblasts cultured for 4 days on untreated and BSP-coated titanium. No statistically significant differences in ALP activity were found between the BSP-treated titanium groups and the untreated titanium control group. Statistically significant differences (p<0.01) compared to the positive or negative control group are indicated by different letters.

ALP initiates the process of osteoid calcification and serves as an early cell marker of osteoblasts. As shown in Fig 7, the positive control group (BMP-7 coating via physisorption) showed the highest ALP activity after 4 days. There was no significant difference in ALP activity between the BSP-coated titanium surfaces and the untreated titanium surface. These findings correlated with our previous in vitro experiments, in which soluble BSP did not affect early ALP activity in hOBs (data not shown). ALP is an early marker of osteoblast differentiation because its expression peaks before the onset of mineralization [72]. Gordon showed that adenovirus-mediated overexpression of BSP led to a significant increase in ALP activity in mouse osteoblasts after three days but no significant difference at day 10 compared to the control treatment [32]. In contrast to the method of Gordon, we did not use βGP and ascorbic acid to enhance early ALP activity. Osteoblast-like MC3T3-E1 cells cultured on BSP-coated plastic previously showed a significant increase in ALP activity after 4 and 5 days [73]. Bovine BSP enhanced ALP activity in rat bone marrow cells with osteoblastic phenotypes that were cultured on a collagen matrix for 3 weeks [74]. This evidence matches the findings of Wang, who implanted BSP collagen into rat calvarial defects and detected the first significant difference in ALP activity between the BSP group and the control group at seven days and observed a peak of ALP activity at 14 days [75]. Our results indicate that BSP coating of titanium does not induce an early increase in ALP activity in primary hOBs.

### Mineralization of the extracellular matrix

**Fig 8:**
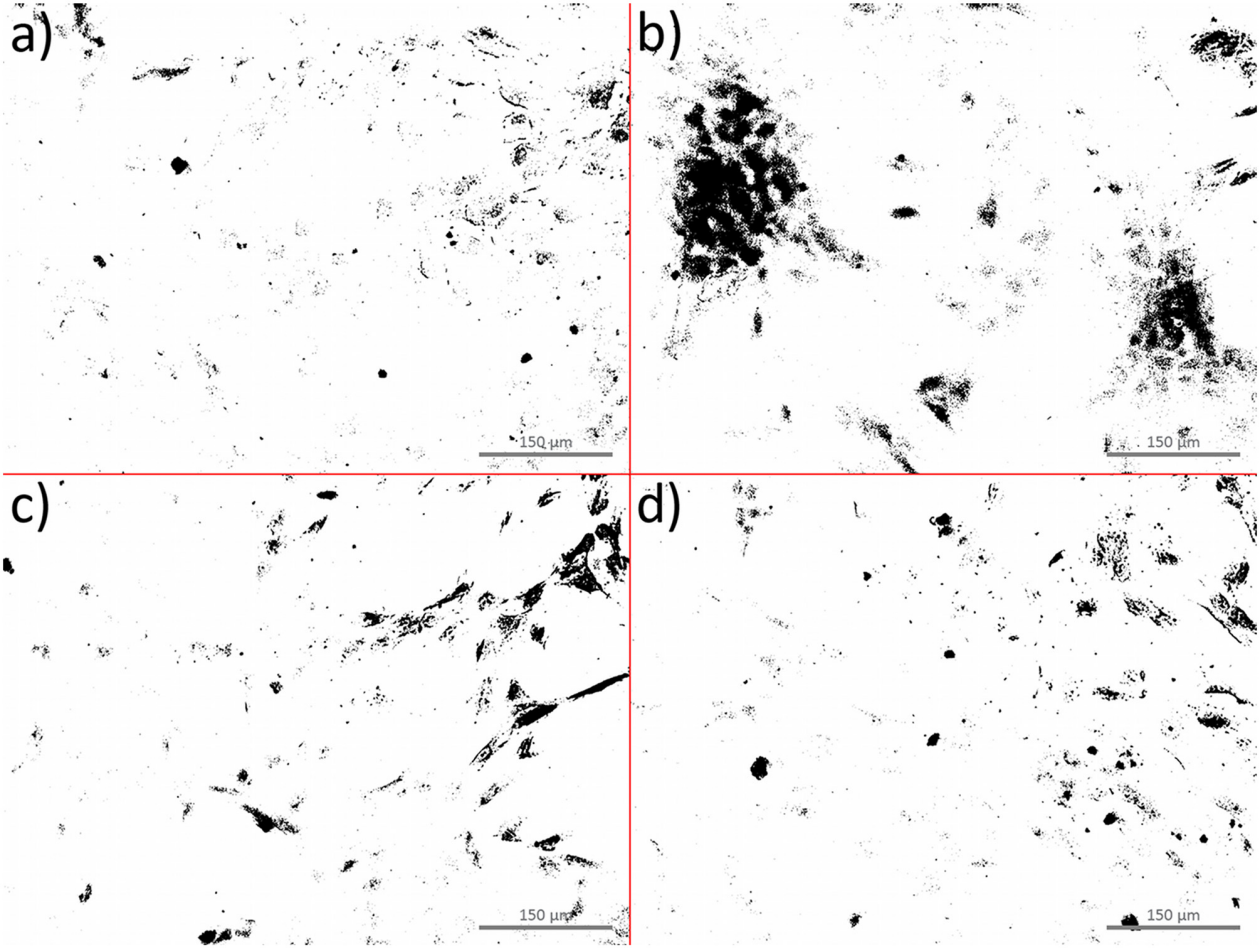
Representative binary images of Alizarin Red S-stained osteoblasts after three weeks in culture. Calcium deposition is depicted by black staining. The ratio of black to white pixels was (a) 1,7 ± 0,5% in cell proliferation medium, (b) 7,9 ± 3,0% in differentiation medium, (c) 3,9 ± 1,5% in medium supplemented with BSP (1 μg/ml) and (d) 3,4 ± 1,4% in medium supplemented with BMP-7 (100 ng/ml).

After three weeks, Alizarin Red S staining revealed increased calcium deposition in the osteoblast populations cultured with additive BSP and BMP-7 compared to the control group with no additives (proliferation media) (Fig 8). One possible explanation for the increase in calcium deposition via BSP despite no change in ALP activity (Fig 7) may be an initial reduction in the osteoblast proliferation rate caused by BSP [32,66]. After a three-week culture period, the proliferative phase ended and confluence was not reached. Thus, the initial inhibitory effect of BSP on the cell proliferation rate no longer affected the cell population, and BSP, known as an indicator of the late stage of osteoblastic differentiation [76], was able to exert its stimulatory effect on mineralization.

### Gene expression

**Fig 9:**
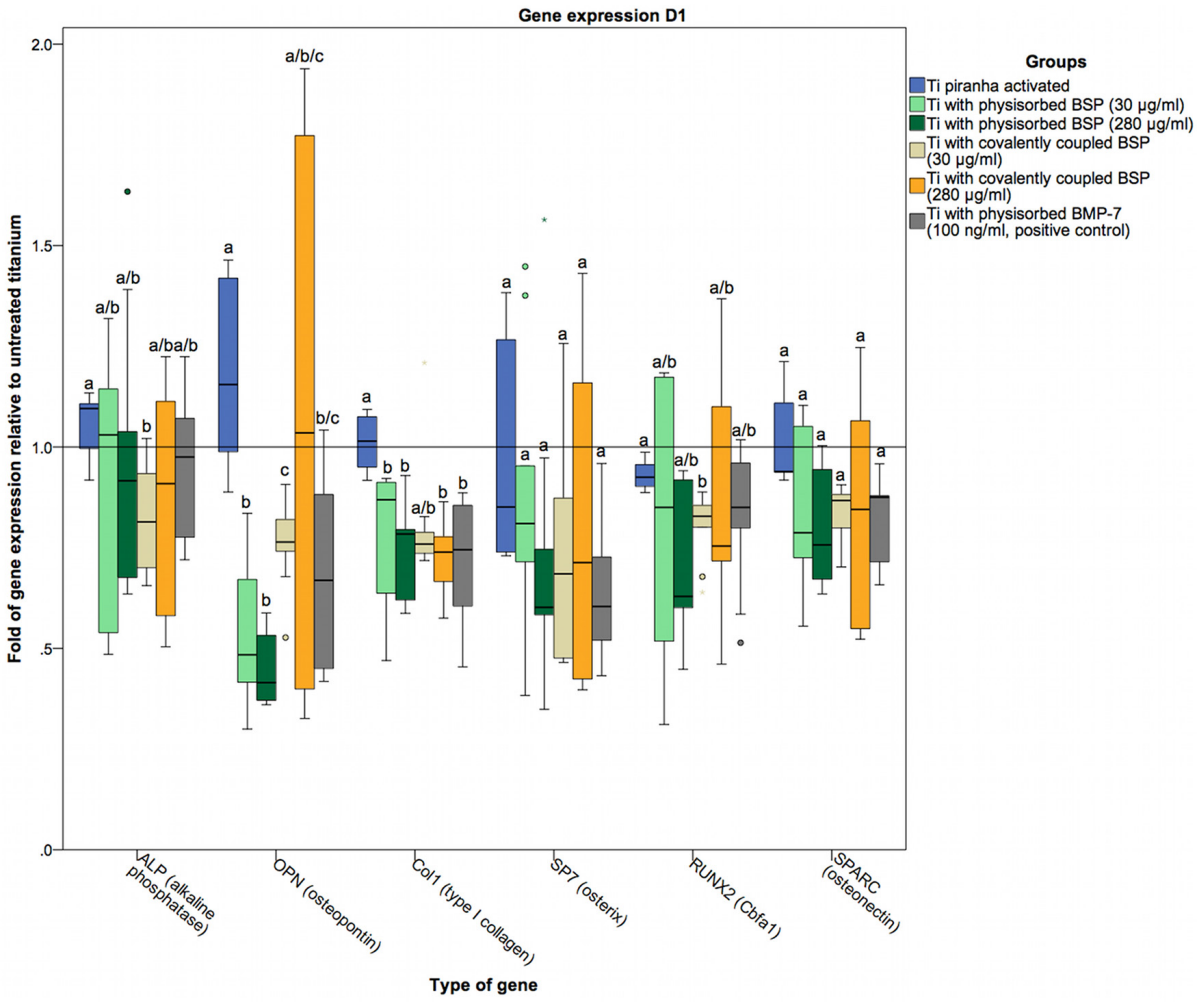
Gene expression in hOBs cultured on titanium on day 1. Data are represented as fold-changes in gene expression relative to hOBs cultured on untreated titanium surfaces. Statistically significant differences (p<0.05) between the groups are indicated by different letters.

**Fig 10:**
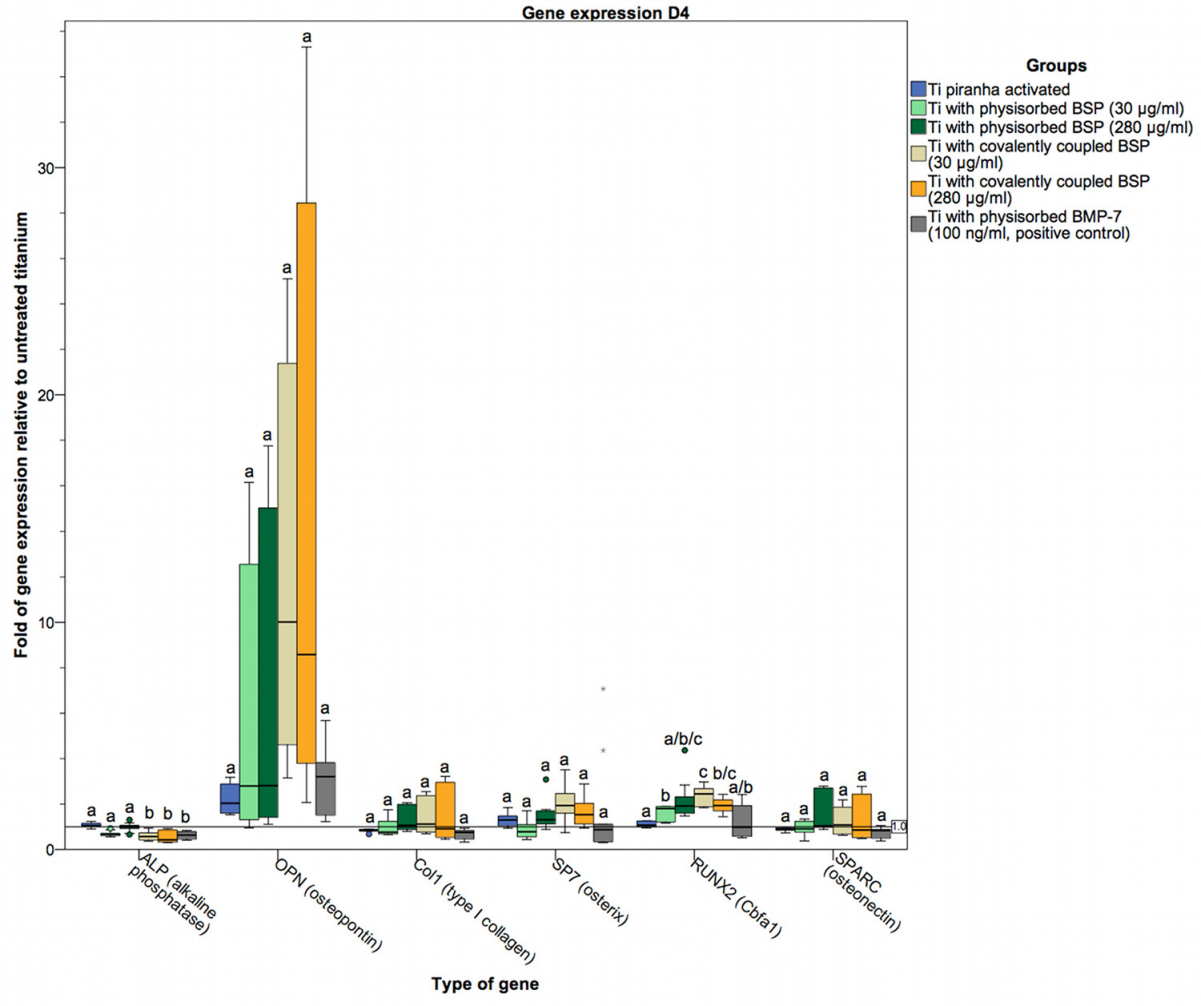
Gene expression in hOBs cultured on titanium on day 4. Data are represented as fold-changes in gene expression relative to hOBs cultured on untreated titanium surfaces. In comparison to day 1, a general trend toward upregulation was observed, and this upregulation was remarkably high for OPN without reaching statistical significance between the coated and uncoated titanium groups. Only RUNX2 was significantly upregulated in all BSP-coated groups (except Ti coated with BSP at 280 μg/ml via physisorption) compared to the uncoated, piranha-activated control group. ALP was slightly downregulated, reaching statistical significance for the groups coated with BSP via covalent coupling and the positive control group compared to the groups coated with BSP via physisorption. Statistically significant differences (p<0.05) between the groups are indicated by different letters.

**Fig 11:**
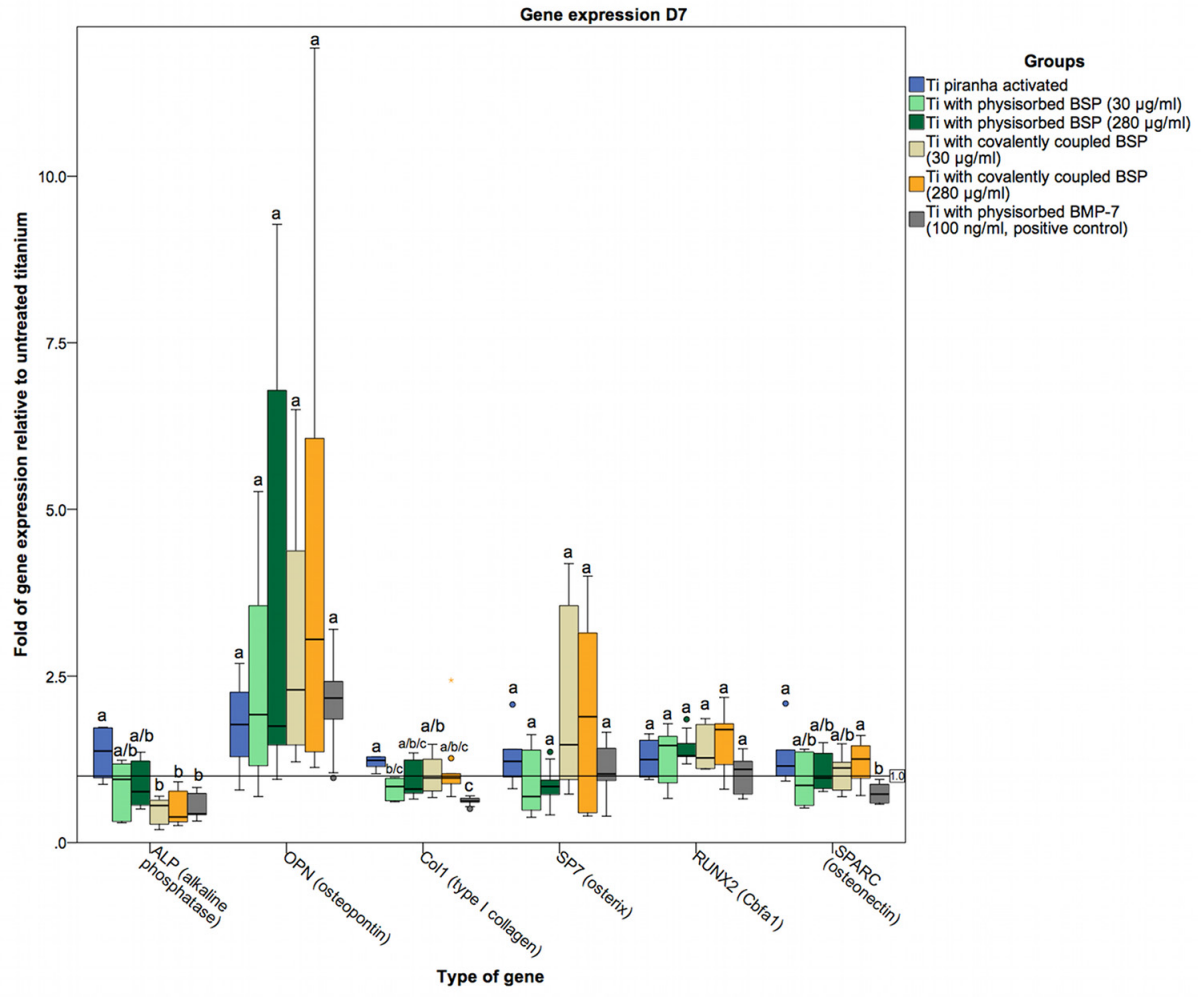
Gene expression in hOBs cultured on titanium on day 7. Data are represented as fold-changes in gene expression relative to hOBs cultured on untreated titanium surfaces. These expression patterns resemble the gene expression patterns detected on day 4, with a statistically significant but small reduction in ALP expression in the groups coated with BSP via covalent coupling and the positive control group and a general nonsignificant tendency toward upregulation of all other genes compared to the uncoated, piranha-activated control group. Statistically significant differences (p<0.05) between the groups are indicated by different letters.

To assess the osteogenic differentiation of osteoblasts cultured on BSP-coated titanium surfaces, the gene expression of specific markers was examined at the RNA level. The markers used were ALP as an early marker of osteoblast differentiation [77]; OPN (a SIBLING family gene), Col1 as a principal organic component of the bone ECM secreted by osteoblasts; the zinc finger transcription factor SP7 (osterix), which is significantly involved in osteoblast differentiation and bone formation [78,79]; the glycoprotein osteonectin (SPARC), which is secreted by osteoblasts during bone formation and initiates mineralization [80]; and RUNX2 (Cbfa1) as an osteoblast-specific transcription factor [81–83]. The gene expression levels in our coated titanium sample groups were compared to those in a positive control group coated with BMP-7 via physisorption. Because titanium is known to enhance the gene expression of RUNX2 and SP7 [84,85], the coated titanium sample groups were also compared to a control group of piranha-activated titanium not subjected to any further treatment. On day 1, the gene expression of ALP in the groups with surface modifications was no different from that in the piranha-activated control group. Only the group of titanium disks coated with BSP at a low concentration (30 μg/ml) via covalent coupling showed a statistically significant reduction, albeit only a marginally decrease, in the expression level of ALP (Fig 9). On days 4 and 7, both groups of titanium disks coated with BSP (30 and 280 μg/ml) via covalent coupling, as well as the BMP-7-treated positive control group, displayed significantly lower ALP expression levels than the uncoated, piranha-activated titanium control group (Figs 10 and 11). On day 1, we found slight difference in OPN and Col1 expression between the groups (Fig 9). However, at subsequent time points, these differences were diminished, with no significant differences in OPN and Col1 expression between the groups (Figs 9–11). In contrast to the significant differences, the relative expression of OPN in hOBs cultured on Ti disks coated with BSP (via covalent coupling and physisorption) was more than 2-fold higher on days 4 and day 7 than in hOBs cultured on untreated titanium, with a maximum difference of 10-fold on day 4 in cells seeded on Ti disks coated with BSP (30 μg/ml) via covalent coupling.

We did not observe any statistically significant differences in the SP7 and SPARC levels, but the expression of RUNX2 was significantly higher in all BSP-coated groups on day 4. However, with increasing confluence of the cell cultures, these differences were completely diminished on day 7 (Fig 11). There was no evidence of a concentration-dependent influence of BSP coating on gene expression. These findings are similar to those of Gordon and Wang. Gordon showed an increase in RUNX2 and SP7 expression after the addition of BSP to primary rat osteoblast cultures. This stimulation was significant after 3 and 5 days but was no longer detectable at day 10 [32]. Wang demonstrated a significant increase in RUNX2 expression at 9 days and in SP7 expression as early as 4 days after BSP-collagen implantation into rat calvarial defects [75].

## Conclusion

Titanium surfaces were activated with piranha solution and coated with BSP via physisorption or covalent coupling. Both methods were effective in binding BSP to the surface, and the resulting surfaces showed no significant difference in cell viability compared to the untreated titanium surface. Cell adhesion on titanium surfaces coated with BSP via physisorption was not different compared to that on untreated titanium surfaces. However, significantly less cell adhesion was observed on titanium coated with BSP via covalent coupling during the first hours in culture, although this reduction normalized after 24 hours. During the first week in culture, the osteoblastic proliferation rate was significantly lower on BSP-treated titanium, particularly on surfaces coated with BSP via physisorption. The spreading of individual cells and the confluence of the hOB population on BSP-coated titanium were reduced during the first five days in culture. Cell migration showed a tendency to be positively influenced by coating of titanium with the higher concentration of BSP. After three or four days in culture, neither a qualitative nor a quantitative difference in ALP activity in the BSP groups was observed. After 21 days, the medium supplemented with BSP led to increased spreading and confluence of the hOB population as well as enhanced calcium deposition compared to the cell growth medium and the differentiation medium. Gene expression, particularly that of osteopontin and RUNX2, was enhanced by BSP coating at day 4, with a trend toward a stronger influence of BSP applied via covalent coupling than via physisorption. In summary, BSP delays the initial proliferative phase but appears to influence the differentiation of osteoblastic cells and mineralization of the ECM via OPN and RUNX2. Considering the impact of the higher BSP concentration on cell migration, this effect of BSP could play a pivotal role in the observed events. However, further studies are needed to determine the value of BSP coating and to evaluate the effects of BSP in an in vivo model. Physisorption is a faster and cheaper method that induces greater initial adhesion, less proliferation and similar effects on gene expression compared to covalent coupling.

## Supporting Information

S1 Fig Table of Contents graphic (TOC). (TIF)

S1 File Abbreviations. (PDF)
