# Supplementary figures and images for: Surface Functionalization of Orthopedic Titanium Implants with Bone Sialoprotein
Source: PLoS One. 2016 Apr 25;11(4):e0153978. doi: 10.1371/journal.pone.0153978 (PMC4844107; doi:10.1371/journal.pone.0153978)

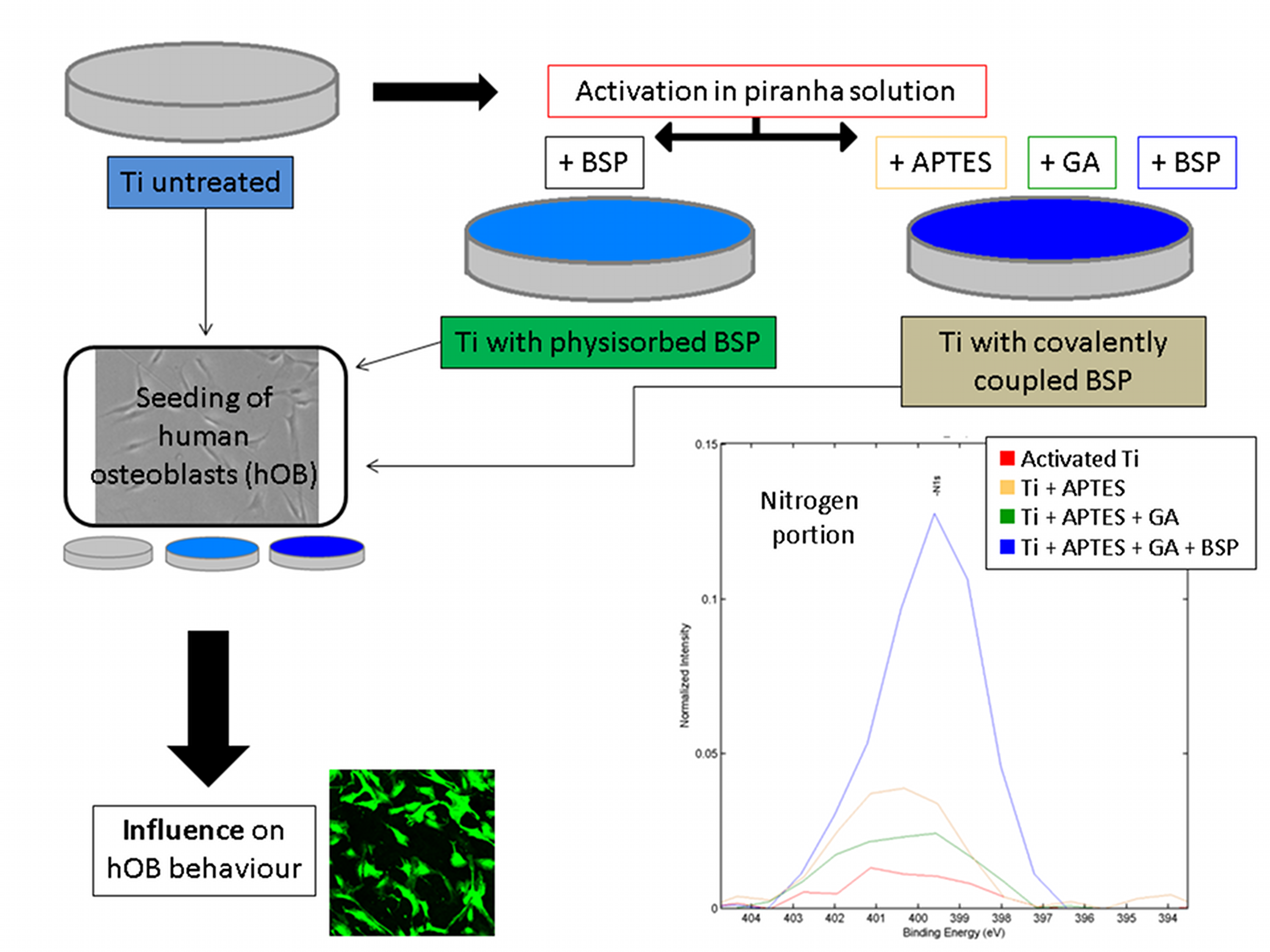

Supplement: S1 Fig — (TIF) [file pone.0153978.s001.tif]
