# Supplementary material for: Surface Functionalization of Orthopedic Titanium Implants with Bone Sialoprotein
Source: PLoS One. 2016 Apr 25;11(4):e0153978. doi: 10.1371/journal.pone.0153978 (PMC4844107; doi:10.1371/journal.pone.0153978)
Supplement: S1 File — (PDF) [file pone.0153978.s002.pdf]

ALP, alkaline phosphatase; APTES, (3-aminopropyl)triethoxysilane; BMP-7, bone morphogenetic protein 7; BSP, bone sialoprotein; BTE, bone tissue engineering; C, carbon; CaP, calcium phosphate; Cbfa1, core-binding factor subunit alpha-1; CLSM, confocal laser scanning microscopy; ECM, extracellular matrix; eGFP, enhanced green fluorescent protein; FCS, fetal calf serum; GA, glutaraldehyde; hOB, primary human osteoblasts; MTT, 3-(4,5-dimethylthiazol-2-yl)-2,5-diphenyltetrazolium bromide; N, nitrogen; NC, nanocomposite; O, oxygen; OPN, osteopontin; PLGA, poly(D,L-lactic-co-glycolic acid); RGD, arginine-glycine-aspartate; RUNX2, Runt-related transcription factor 2; Si, silicon; SP7, osterix (SP7) gene; SPARC, secreted protein acidic and rich in cysteine; Ti, titanium; TICER, titanium with hydroxyapatite ceramic; XPS, x-ray electron spectroscopy; ZDEC, zinc diethyldithiocarbamate.
